# A Membrane Magnetoelastic Generator for Acoustic Energy Harvesting

**DOI:** 10.1002/advs.202409063

**Published:** 2025-04-25

**Authors:** Ziyuan Che, Jing Xu, Xiao Wan, Chrystal Duan, Jun Chen

**Affiliations:** ^1^ Department of Bioengineering University of California, Los Angeles Los Angeles CA 90095 USA

**Keywords:** acoustic energy harvesting, clean energy, magnetoelastic effect, soft system

## Abstract

Acoustic waves are a type of energy propagation through a medium with a low energy density, which makes it challenging to be converted into electricity. Here, a membrane magnetoelastic generator is presented as a fundamentally new technology for acoustic energy harvesting that is centered on a coupling of magnetoelastic effect and electromagnetic induction. It is as thin as 400 µm, holds a skin‐alike modulus of 2.59 × 10^7^ Pa, a low internal impedance of 137 Ω, and a high current density of 107 mA m⁻^2^ at a sound pressure level (SPL) of 95 dB. It can efficiently harvest acoustic energy to charge a 47 µF commercial capacitor to 2 V in 20 s. With an all‐in‐one‐body design, the membrane magnetoelastic generator shows stable electrical output against environmental moisture and particulates. Toward practical application, it is demonstrated to harvest acoustic energy from a vehicle stereo and use recycled energy to charge a smartphone. Its inherent water‐/dust‐proof features open new possibilities for acoustic energy harvesting from city buildings, highways, airplanes, and even battlefields.

## Graphical abstract

This study introduces a thin, flexible, and water‐/dust‐proof membrane magnetoelastic generator that converts acoustic energy into electricity through the coupling of giant magnetoelasticity and electromagnetic induction. Featuring a skin‐like modulus, low impedance, and high output current density, this fundamentally new platform technology can efficiently harvest sound energy from urban noises, transportation, and electronic devices, offering a sustainable power solution for various applications.

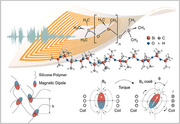

## 1 Introduction

In an era where sustainable energy sources are of paramount importance, the exploration of novel and efficient energy harvesting techniques to harness renewable energy resources has become a critical scientific endeavor.^[^ ^1^, ^2^, ^3^ ^]^ Acoustic energy harvesting, a field that has witnessed substantial progress in recent times, stands as a promising contender.^[^ ^4^, ^5^ ^]^ This technology capitalizes on the energy from ambient noise and vibrations, transforming it into usable electrical power.^[^ ^6^, ^7^, ^8^ ^]^ It holds the potential to redefine how we power small electronic devices, especially in scenarios where sound waves are abundant while conventional energy sources are either impractical or inaccessible. Acoustic energy harvesting could be a transformative force across various sectors.^[^ ^9^, ^10^, ^11^, ^12^ ^]^ However, the journey toward acoustic energy harvesting is fraught with challenges. One of the main challenges lies in the relatively low energy density of sound waves,^[^ ^13^, ^14^ ^]^ especially compared to other renewable energy like solar or wind power in the ambient environment.^[^ ^15^ ^]^

Current mechanisms for acoustic energy harvesting are predominantly based on piezoelectric, electrostatic, electromagnetic, and triboelectric effects.^[^ ^7^, ^16^, ^17^ ^]^ The widespread adoption of these working mechanisms is largely challenged by the expensive materials,^[^ ^18^ ^]^ complex structures,^[^ ^19^, ^20^, ^21^ ^]^ high inner impedance,^[^ ^22^ ^]^ low current output,^[^ ^23^ ^]^ and vulnerability to the humidity and the dust.^[^ ^6^, ^7^, ^24^, ^25^ ^]^ Among these platform technologies, triboelectric effect‐based acoustic energy harvesting is the most recent invention.^[^ ^6^, ^7^ ^]^ Relying on the surface triboelectrification between two dissimilar materials, the triboelectric effect‐based acoustic energy harvesting is vulnerable to moisture and particulates,^[^ ^26^ ^]^ although many acoustic resources in nature exist in such an environment. Moreover, this material friction‐based working mechanism easily induces material wear^[^ ^27^ ^]^ and results in performance degradation over time.^[^ ^28^ ^]^

Therefore, there is a pressing need to develop a cost‐effective, water‐/dust‐proof, durable acoustic energy harvesting device with high current output and low internal impedance. Recent developments, such as the integration of gradient‐index phononic crystals (GRIN‐PCs) and Helmholtz resonators, have shown promising results in overcoming these challenges,^[^ ^29^, ^30^ ^]^ by focusing and amplifying sound energy to enhance harvesting efficiency. To further enhance acoustic energy harvesting efficiency, several studies have explored the use of 3D‐printed phononic crystals and dual‐tube Helmholtz resonators. These approaches allow for a more controlled focusing of sound waves, improving the energy density at the location of the harvester. For instance, studies have demonstrated that a dual‐tube resonator can increase voltage output by up to 83% and improve operational efficiency across a broader frequency range.^[^ ^31^, ^32^ ^]^ But the predominant challenge of water and dust proof still requires further investigation into the material and mechanism used for acoustic energy harvesting.

In this paper, we introduce a fundamentally new platform technology for acoustic energy harvesting based on the recently discovered magnetoelastic effect in soft matter by our group,^[^ ^33^ ^]^ which has been harnessed to develop magnetoelastic generators (MEG) in earlier studies via a coupling of magnetic induction (MI).^[^ ^33^, ^34^ ^]^ They were shown to efficiently convert mechanical energy into electricity with high current output and low internal impedance.^[^ ^34^ ^]^ Herein, with a rationally designed array‐stack structure and a thickness of 400 µm, the membrane MEG has a skin‐like Young's modulus of 2.59 × 10^7^ Pa and can harvest acoustic energy across a wide frequency range from 20 to 1400 Hz, covering the most ambient sound frequency range in daily life. Utilizing a Helmholtz cavity, the as fabricated magnetoelastic generator can produce an output current density of 107 mA m⁻^2^ at a Sound Pressure Level (SPL) of 95 dB with a low internal impedance of 137 Ω. By incorporating a rectifier, we demonstrate the device's ability to charge a 47 µF commercial capacitor to 2 V in just 20 s. Furthermore, we demonstrated that it could harness long‐ignored acoustic energy from a car stereo to charge a smartphone. The concept and design presented in this work can be adapted to various acoustic energy harvesting scenarios and are intended to stimulate further research and innovation in this field. With a collection feature of being cost‐effective, intrinsically waterproof, stable against particulates, low internal impedance, high current output density, the magnetoelastic generator is a milestone invention in the acoustic energy harvesting community.

## 2 Results and Discussions

### 2.1 Working Principle

**Figure 1:**
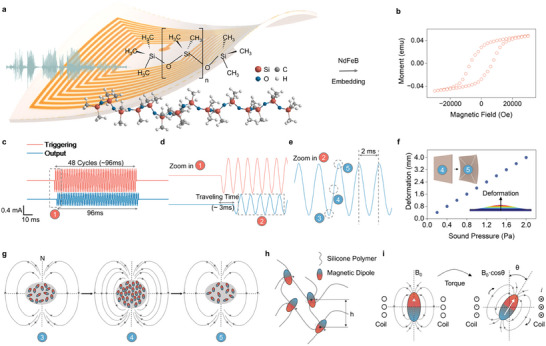
Membrane magnetoelastic generators for acoustic energy harvesting. a) Schematic of the membrane MEG‐based acoustic energy harvester utilizing PDMS substrates. b) Hysteresis loops characterizing the membrane MEG‐based acoustic energy harvester. c) Depiction of the triggering sound and the membrane MEG's response waveform at a frequency of 500 Hz; the signal persists for 96 ms, encompassing 48 cycles. d) A magnified view of the triggering and response waveforms, highlighting a 3 ms delay, which represents the sound's travel time through the air. e) Close‐up of the membrane MEG ’s response waveform; within a 2 ms cycle, the membrane's deformation aligns with the phase of the traversing sound wave. f) Membrane deformation under varying sound pressures, illustrating a linear trend that increased sound pressure results in greater deformation. g) Diagram depicting various states of the membrane MEG in relation to soundwave‐induced deformation. The associated magnetic dipole arrangements are evident: the film transitions from a structured magnetic dipole alignment to a more scattered configuration when deformed. h) Legend detailing the silicone polymer and its associated magnetic dipoles, including the inter‐dipole distances. i) The magnetic particle experiences a torque, denoted as *θ*, due to the deformation exerted on the polymer.

For harvesting acoustic energy, **Figure** **1**a depicts a thin, flexible, waterproof acoustic energy harvester based on the magnetoelastic effect with a polydimethylsiloxane (PDMS) substrate. The membrane magnetoelastic generator comprises two layers: the magnetomechanical coupling (MC) layer, which facilitates the conversion from mechanical to magnetic energy, and the MI layer responsible for magnetic to electrical conversion. The MC layer is crafted from magnetoelastic materials that blend PDMS with magnetic nanoparticles. In this study, NdFeB (Neodymium‐iron‐boron) serves as the magnetic nanoparticles, as its single crystal structure shown in Figure S1 (Supporting Information). A photo of the well‐fabricated MC layer is presented in Figure S2 (Supporting Information) with a scanning electron microscope (SEM) image to show its inner microstructure (Figure S3, Supporting Information). The magnetic hysteresis loop of the MC layer is demonstrated in Figure 1b. Paired with the MI layer, constructed from a soft copper yarn ≈67 µm in diameter, the system is aptly designed for acoustic energy harvesting. Sound, a type of mechanical wave, conveys energy that moves through a medium via particle vibrations. As depicted in Figure S4 (Supporting Information), the mechanical wave induces a deformation in the device when a sound wave traverses the membrane MEG. During this process, the MC layer converts the mechanical energy into the magnetic field variation, and the MI layer subsequently produces a Lorentz current, competing the cycle of acoustic wave to electricity conversion.

A hallmark of this system (MC layer) is its foundation on the magnetoelastic effect in soft magnetic systems. The magnetoelastic effect, which denotes a shift in the magnetic flux density of a ferromagnetic material due to externally applied mechanical stress, was identified in the mid‐19th century.^[^ ^35^ ^]^ It has been observed in rigid metals and metal alloys such as Fe1‐xCox,^[^ ^36^ ^]^ TbxDy1‐x Fe_2_ (Terfenol‐D),^[^ ^37^ ^]^ and GaxFe1‐x (Galfenol).^[^ ^38^ ^]^ In 2021, the giant magnetoelastic effect was discovered in a soft composite system.^[^ ^33^ ^]^ This discovery was first incorporated into the system we present for acoustic energy harvesting. A comprehensive illustration is provided in Figure 1c, where the electrical output of the device responds to an ambient triggering acoustic wave. Currents are generated in the presence of the triggering acoustic wave and cease when it is removed. The magnified electrical output waveform in Figure 1c distinctly reveals that the generated acoustic wave mirrors the frequency of the electrical signal, albeit with a delay of ≈3 ms. This delay corresponds to the sound wave's travel time from its source to the membrane MEG. Moreover, as depicted in Figure 1e, during the 2 ms cycle of the 500 Hz wave, the MC layer membrane undergoes repeated deformations as the sound wave consistently passes through. This results in continuous changes in magnetic flux density, producing a steady electrical output that aligns with the sound wave prompting the membrane's deformation. With increased sound pressure, more pronounced membrane deformations can be observed, as shown in Figure 1f.

The energy conversion via the pronounced magnetoelastic effect in soft elastomers is explicable at both micro and atomic scales. At the microscale, compressive stress on the soft polymer composite induces a corresponding shape deformation, leading to magnetic particle‐particle interactions (MPPI). This includes alterations in the distance and orientation of inter‐particle connections. Membrane deformation under pressure results in minor changes in magnetic density. As illustrated in Figure 1g, the system's magnetic density varies in line with the phase of the sound waveform as it moves through the membrane, causing corresponding deformations. The variation in the magnetic field is quantified in Figure S5 (Supporting Information), which plots magnetic flux density against stress. For all samples, the magnetic flux density of the films diminishes as stress intensifies. Magnetic nanoparticles are perceived as singular dipoles aligned in the impulse magnetic field. Specifically, as depicted in Figure 1h, external deformation alters the distance between each dipole, modifying the overall magnetic field of the soft system. On the atomic scale, mechanical stress also triggers magnetic dipole‐dipole interactions (MDDI), leading to the rotation and movement of magnetic domains within the particles. As shown in Figure 1i, a torque is applied to each nanomagnetic nanoparticle, and the shift in angle *θ* results in changes in magnetic flux density.

### 2.2 Parameters Optimization

**Figure 2:**
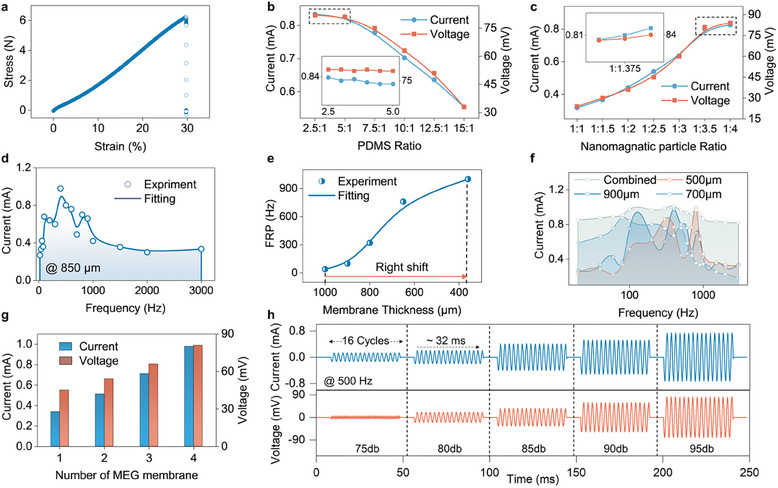
Parameters optimization for magnetoelastic generators. a) The stress‐strain relationship for the membrane MEG. b) The membrane MEG's output current and voltage across varying PDMS ratios; an inset illustrates the output for ratios between 2.5:1 and 5:1. c) The membrane MEG's output current and voltage at different concentrations of nanomagnetic particles; an inset highlights the output for concentrations ranging from 1:3.5 to 1:4. d) The output current from the membrane MEG, with a thickness of 850 µm, spanning frequency ranges of 20–3000 Hz. e) Identification of the FRP for membrane MEGs of varying thicknesses. f) Output current from the membrane MEG at thicknesses of 500, 700, and 900 µm, as well as a combined three‐layer configuration, across a frequency range of 20–1400 Hz. g) The membrane MEG system's output current and voltage in relation to the number of membrane MEG layers incorporated. h) The waveform representing the current and voltage produced by the membrane MEG system under a consistent sound wave frequency of 500 Hz, but at varied Sound Pressure Level (SPL)s.

To enhance the acoustic energy conversion efficiency of the membrane MEG, we conducted a series of systematic investigations on the designing parameters before assembling the components for performance testing. The stress‐strain relationship of the device is illustrated in **Figure** **2**a. Remarkably, the device exhibits Young's modulus reminiscent of human skin, measuring at 2.59 × 10^7^ Pa, and can endure a maximum strain of 130%. For wearable and implantable bioelectronics, it is imperative that the membrane MEG is not only flexible, but also adheres comfortably and safely to skin and organ surfaces.^[^ ^39^, ^40^ ^]^ The setup for output testing is detailed in Figure S6 (Supporting Information), which will be referenced for subsequent current and voltage output tests. Figure 2b explores the impact of varying the PDMS ratio between the elastomer base and the curing agent on the device's energy harvesting efficiency. As the ratio increases, indicating a softer energy harvesting membrane, there is a noticeable decline in both the current and voltage output. This trend suggests that stiffer membranes are more readily deformed by acoustic waves, whereas softer membranes exhibit a reduced deformation degree under identical sound pressures. A closer examination reveals that the output stabilizes when the ratio ranges from 5:1 to 5:2. Balancing this with stretchability, we selected a 5:1 PDMS ratio for this study. Figure 2c delves into the influence of nanomagnetic particle concentration on energy output. As anticipated, increasing the concentration of magnetic nanoparticles within the PDMS substrate leads to heightened energy outputs. This trend can be attributed to the fact that a greater concentration of magnetic nanoparticles results in more significant magnetic flux changes upon deformation. However, this increase is in both current and voltage output plateaus when the magnetic nanoparticle ratio is between 1:3.5 and 1:4. For our research, we opted for a 1 (PDMS) to 4 (magnetic nanoparticle) mass ratio.

Having established the magnetic material parameters, we proceeded to assess how the membrane's physical shape and structure influence energy harvesting efficiency. Our experiments revealed that, for a given membrane thickness, the device's output fluctuates with sound frequency variations. Figure 2g presents a current‐frequency plot for an 850 µm thick membrane, highlighting several resonance points where the output surpasses neighboring frequencies. Figure S7 (Supporting Information) offers a more granular view of these resonance points across three distinct frequencies, underscoring the variance in current amplitude, which peaks at the resonance point. Given the presence of multiple resonance points for each thickness, we focused on the first resonance point (FRP) for in‐depth analysis. The circuit depicted in Figure S8 (Supporting Information) was employed to capture outputs across frequencies with greater precision. As shown in Figure 2e, thinner membranes shift the FRP toward higher frequencies. Capitalizing on this observation, we assembled an array by stacking several membranes of varying thicknesses. Figure S9 (Supporting Information) illustrates this array's structure, with Figure 2f documenting its output. While each thickness has its distinct current performance curve, the combined output remains consistently high across a 20–1400 Hz frequency range, encompassing the majority of everyday acoustic energy. Figure S10 (Supporting Information) provides a clearer representation of the current output for individual and combined thicknesses across various frequencies. Subsequent tests, as shown in Figure 2g, evaluated the average current output based on the number of MC layers in the array. As layer count increases, the device yields progressively higher and more stable output, peaking at a current density of 107 mA m⁻^2^. Last, Figure 2h presents the current and voltage outputs under varying SPL at a consistent 500 Hz frequency. The results clearly indicate a direct correlation: higher sound pressures produce higher current and voltage outputs. And the responding waveform stays unattenuated at all SPL points with consistent frequencies.

### 2.3 Acoustic Energy Harvesting Standard Evaluation

**Figure 3:**
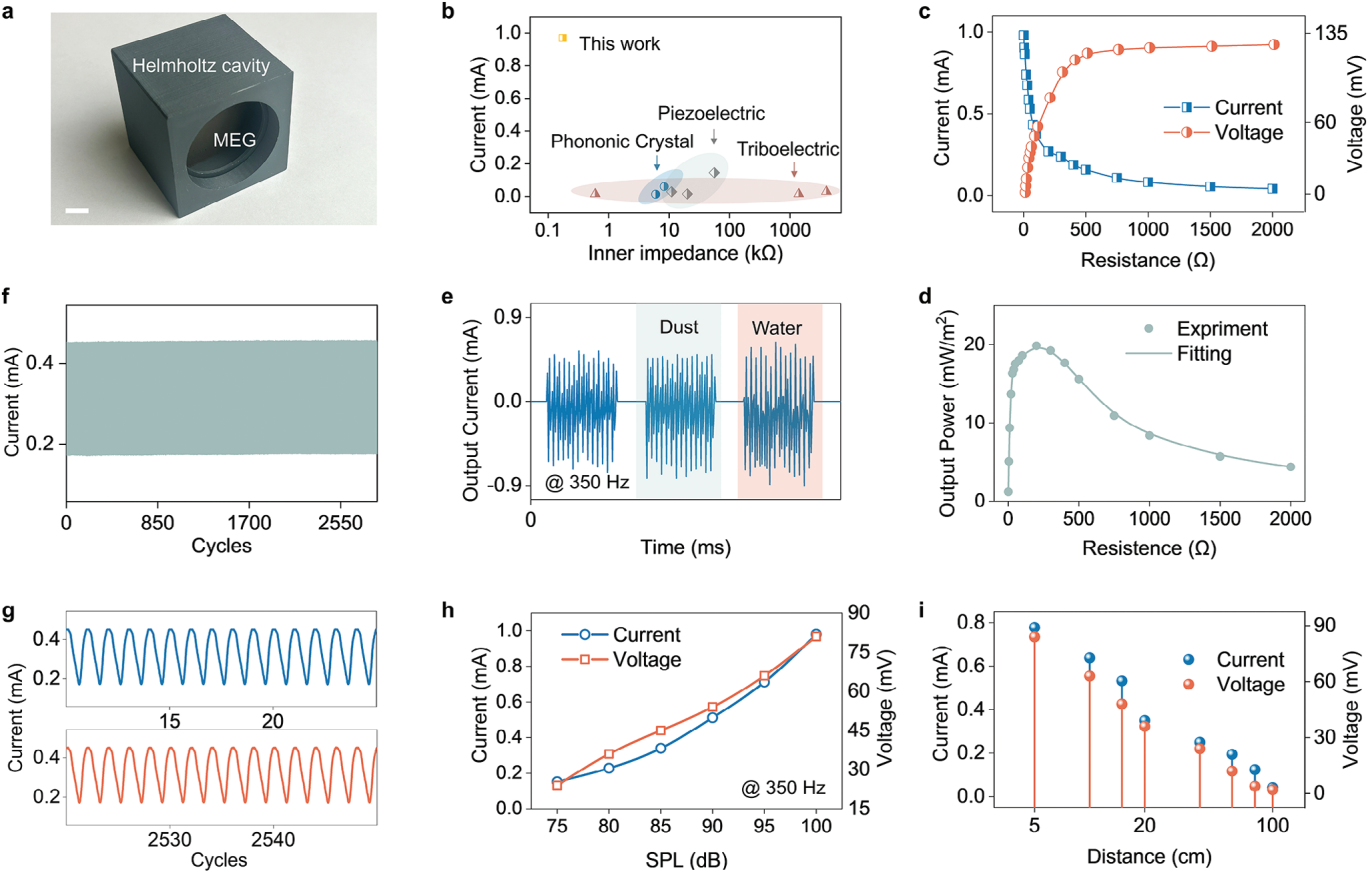
Performance evaluation of the membrane MEG for acoustic energy harvesting. a) Image of the membrane MEG array positioned within a Helmholtz cavity. Scale bar, 1 cm. b) A comparison of the current produced by the acoustic energy harvester in this study versus prior phononic crystal,^[^ ^29^, ^30^ ^]^ triboelectric^[^ ^7^, ^16^, ^17^ ^]^ and piezoelectric^[^ ^40^, ^41^, ^42^ ^]^ methods. c) The external resistances' output voltage and current, and d) power generated by the membrane MEG from acoustic vibration energy. e) A comparison of current production from the generator device before and after dust and water exposure, demonstrating its waterproof and dustproof capabilities. The consistent output underscores the device's potential for diverse real‐world applications. f,g) Device durability assessment over 2 700 continuous cycles at a frequency of 500 Hz, showcasing the overall waveform from cycles 1–2700 (f). An expanded view captures the initial 10–25 cycles and the 2 525–2 545 cycles (g). h) The device's output current and voltage across varying SPLs. i) The device's output current and voltage as a function of its distance from the sound source.

After meticulously fine‐tuning the parameters and optimizing the structural design of the membrane MEG, we proceeded with performance evaluation for acoustic energy harvesting. For this, we incorporated a Helmholtz cavity to augment energy collection efficiency, as depicted in **Figure** **3**a. Figure S11 (Supporting Information) provides a more comprehensive illustration of the testing setup, showcasing how sound waves generated by a loudspeaker traverse the air and are subsequently captured by the Helmholtz cavity. The overarching testing methodology for the subsequent experiments in this section is delineated in Figure S12 (Supporting Information). Utilizing the Helmholtz cavity, the device achieved a peak current density of 107 mA m⁻^2^. This performance is notably superior to previous acoustic energy harvesting methods that employed triboelectric and piezoelectric materials, as highlighted in Figure 3b. A thorough comparison with prior research is available in Table S1 (Supporting Information). Regarding the comparison between current and voltage, it is important to note that while TENGs and PENGs typically achieve higher voltage outputs, this is often associated with high internal impedance, making it difficult to directly apply these technologies to power small electronics efficiently^[^ ^41^, ^42^ ^]^. In contrast, MEG delivers high current density with low internal impedance (137 Ω), which is advantageous for practical applications requiring direct power delivery to low‐power devices, as it avoids the need for complex power management circuits.

To assess the power delivery of the membrane MEG, we gauged its output performance with external loading resistance. Figure 3c reveals an inverse relationship between the output current and the loading resistance: as resistance escalates, the output current diminishes while the output voltage surges. Beyond a resistance threshold of ≈500 Ω, both the rise in output voltage and the decline in output current stabilize, exhibiting minimal fluctuations. Based on the recorded current and voltage metrics, we determined a peak power output of 4 mW m⁻^2^ at a matched loading resistance of 137 Ω, as shown in Figure 3d. We then evaluated the device's water‐resistant and dust‐resistant capabilities by spraying water and dust directly onto the membrane MEG, as demonstrated in Figure 3e. Maintaining a consistent frequency of 350 Hz, the device exhibited no discernible attenuation post water and dust exposure, attesting to its robust water and dust resistance. This attribute is particularly advantageous for ambient acoustic energy harvesting, where moisture and dust commonly exist. The durability of membrane MEG was evaluated by 2 700 continuous deformation cycles (Figure 3f), and there was no noticeable degradation in current output throughout rigorous testing (Figure 3g). In Figure 3h, we analyzed the device's performance under varying SPLs. The results indicated a quasi‐linear relationship: as sound pressure amplified, both current and voltage outputs correspondingly increased. Last, Figure 3i explores the device's output in relation to the distance separating the sound source and the energy harvester. As anticipated, the output diminishes as the distance expands. However, even at a distance of 1 m, the device retains functionality, producing 50% of its maximum output when positioned 20 cm away from the sound source.

### 2.4 Practical Applications

**Figure 4:**
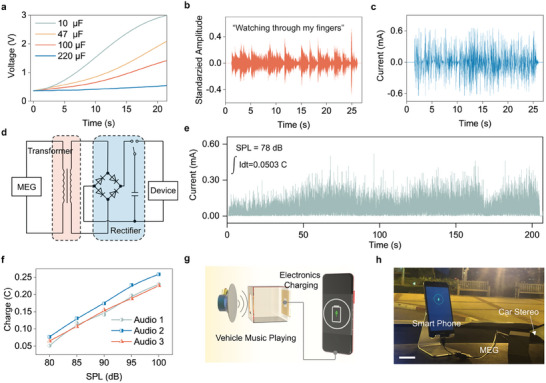
Practical acoustic energy harvesting with ambient moisture and dust. a) Charging trajectory of commercial capacitors powered by the membrane MEG. b) Sound waveform emitted by the commercial loudspeaker, featuring the song “Good Grief” by Bastille, starting at the 30‐s mark with the line “Watching through my fingers”. c) The membrane MEG's generated current waveform corresponding to the sound. d) Schematic representation of the membrane MEG's self‐powered system. e) The membrane MEG circuit's rectified current output; the song, spanning 200 s, accumulated a total charge of 0.0503 C at 78 dB. f) Accumulated charge in relation to SPLs across three different music tracks. g) Depiction of the energy harvesting system designed to power a smartphone. h) A photographic representation of the scenario where the smartphone is powered by acoustic energy, harnessed from the car stereo by the membrane MEG. Scale bar, 10 cm.

To demonstrate the membrane MEG device for real‐world acoustic energy harvesting, we utilized it to charge capacitors of varying capacitance with a loudspeaker, as illustrated in **Figure** **4**a. The sound waves were generated by the computer connecting to a rectifier, and subsequently, a loudspeaker. The output of the rectifier ensures the loudness of the sound, and the computer plays the pre‐recorded noise sample. For charging experiments, the membrane was put at 10 cm to the loudspeaker with the SPL at which distance is 80 dB. The commercial capacitors tested included values of 10 µF and 47 µF. Remarkably, these capacitors were charged to 3 V and 2 V in just 20 s, respectively. Additionally, capacitors with higher capacitance values, 100 µF and 200 µF, achieved a charge of 1 V within 30 and 60 s, respectively. For a more practical assessment, we examined the energy harvester's proficiency in capturing acoustic energy from a commercial loudspeaker. Figure 4b showcases the sound waveform emitted by the loudspeaker, while Figure 4c presents the corresponding current generated by the membrane MEG. A discernible correlation between the current waveform and the music is evident: louder sound segments correspond to higher current peaks, and when the sound ceases, the current generation halts as well. To facilitate the charging of electronic devices, the acoustic energy‐derived electricity was channeled to a management circuit, employing a commercial capacitor as an energy storage medium, as depicted in Figure 4d. Figure 4e displays the rectified signal harvested by the membrane MEG from a loudspeaker playing an entire song. At an SPL of 78 dB, a song spanning 200 s yielded ≈0.05 C of charge via the membrane MEG. We further evaluated the energy generation performance using three distinct songs played at varying volume levels, as shown in Figure 4f. Across all three audio tracks, a clear trend emerges: the total charge generated increases proportionally with the SPL, operating effectively within the typical SPL ranges encountered in daily life. In addition, we conducted a test depicted in Figure 4g, where the device was positioned in a running car. As the car's stereo played music, the membrane MEG captured and recycled the acoustic energy to charge a smartphone. Figure S13 (Supporting Information) provides a visual representation of this energy harvesting setup. A more detailed view is presented in Figure 4h, where the membrane MEG is strategically placed adjacent to the car's stereo, harnessing the sound wave of music to power the smartphone. Movie S1 (Supporting Information) offers a dynamic view of the smartphone charging process powered by the membrane MEG while the car is in motion. Through these comprehensive tests, we have effectively demonstrated the membrane MEG ’s practical viability for ambient acoustic energy harvesting with significant promise, offering a sustainable energy solution for powering electronic devices.

## 3 Conclusion

In this work, we developed a flexible, thin, water‐/dust‐proof acoustic energy harvester based on the magnetoelastic effect in a soft material system. We have tested and confirmed several attractive features of the MEG, including a skin‐alike modulus of 2.59 × 10^7^ Pa, a low internal impedance of 137 Ω and a high current density of 107 mA m⁻^2^ at a SPL of 95 dB. It can efficiently harvest acoustic energy, charging a 47 µF commercial capacitor to 2 V in just 20 s. Additionally, combining MC layer with different thicknesses enables a wide frequency range of 20–1400 Hz acoustic energy harvesting. We have also demonstrated the feasibility of the device recycling acoustic energy from a car stereo to charge a smartphone. Our device offers a practical solution for ambient acoustic energy harvesting, especially where moisture and dust commonly exist, like highways and city buildings.

## 4 Experimental Section

### Fabrication of the Membrane MEGs

The neodymium‐iron‐boron (NdFeB, Magnequench) magnetic powder was evenly mixed with polydimethylsiloxane substrate (PDMS, Sylgard 184) at a mixing ratio of 4:1. The weight ratio of the magnetic powder and PDMS was measured to be 4:1. Next, the as‐prepared magnetic paste was poured into a 3D‐printed mold (polylactic acid, PLA) of 30 by 30 by 1 mm (length, width, height, height varies with different membrane thickness) and transferred to an oven set at 70 °C for over 4 h. The cured MC layer was then removed from the mold and magnetized by an impulse magnetizer (IM‐10‐30, ASC Scientific) with an impulse field.

### Fabrication of the Membrane MEG‐Based Acoustic Energy Harvester

The Helmholtz cavity was 3D printed by a 3D printer (Ender‐3, Ender. Inc.) with PLA. And the spacer used to stack each layer of MEG together was cut with a laser cutter (ULTRA R5000, Universal LaserSystem) on acrylics (thickness 1 mm). And the as‐fabricated membrane MEG was assembled with the cavity and the spacer.

### Electrical Performance Measurement

The device's current signal was recorded using a Stanford low‐noise current preamplifier (model SR570). Meanwhile, its voltage signal was captured with a Stanford voltage preamplifier (model SR560). For standardized testing, a flat plate was employed, which exceeded the dimensions of the membrane MEG, and it was interfaced with an electrodynamic shaker system. This system comprised a function generator (AFG1062, Newark), a linear power amplifier (PA‐151, Labworks Inc.), and an electrodynamic transducer (ET‐126HF, Labworks Inc.). To facilitate capacitor charging, the harvested electricity underwent processing via a diode bridge rectifier (MBSK16SE) and was subsequently channeled through a toroidal transformer.

## Conflict of Interest

The authors declare no conflict of interest.

## Author Contributions

Z.C., J.X., and X.W. contributed equally to this work. J.C. initialized and supervised the project. J.C., Z.C., and X.W. performed conceptualization. Z.C. and X.W. designed the experiments. T.H., N.N., D.N., and T.Z. assisted in conducting experiments and device performance testing. J.C., Z.C., and J.X. designed and created the figure. Z.C. wrote the original manuscript. All the authors reviewed and made technical comments on the manuscript.

## Supporting information



Supporting Information

Supporting Information Movie 1

## Data Availability

The data that support the findings of this study are available from the corresponding author upon reasonable request.
