# Supplementary material for: A Membrane Magnetoelastic Generator for Acoustic Energy Harvesting
Source: Adv Sci (Weinh). 2025 Apr 25;12(20):2409063. doi: 10.1002/advs.202409063 (PMC12120712; doi:10.1002/advs.202409063)
Supplement: Supplementary file 1 — Supporting Information [file ADVS-12-2409063-s002.docx]

**Supporting Information**

**A Membrane Magnetoelastic Generator for Acoustic Energy Harvesting**

*Ziyuan Che, Jing Xu, Xiao Wan, Chrystal Duan, Jun Chen^,^**

Z. Che, J. Xu, X. Wan, C. Duan, Prof. J. Chen

Department of Bioengineering

University of California, Los Angeles

Los Angeles, California 90095, USA

Email: [jun.chen@ucla.edu](mailto:jun.chen@ucla.edu) (J. C.)

**The PDF file includes:**

Figure S1 to S13

Table S1

**Other Supporting material for this manuscript includes the following:**

Movie S1


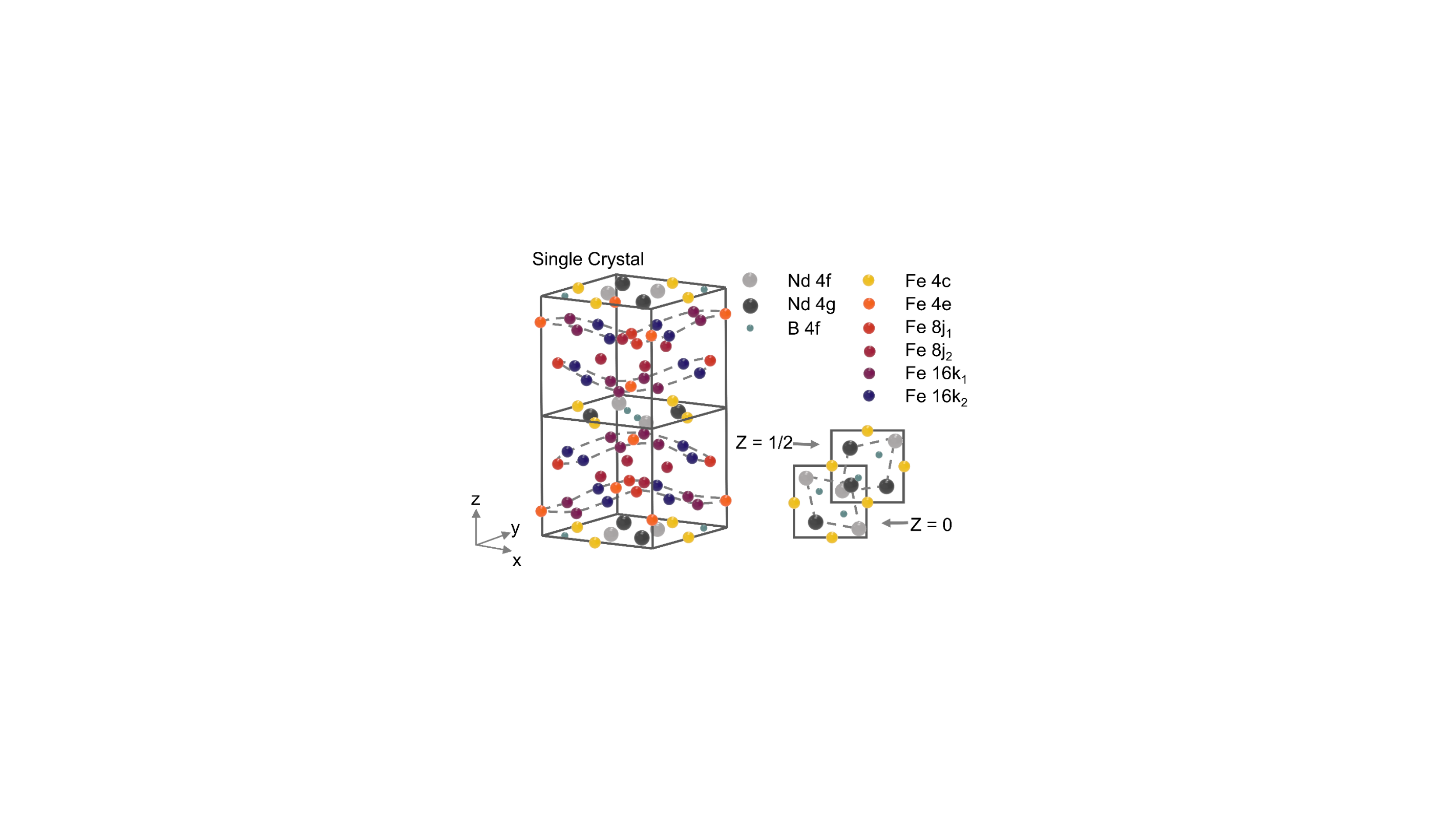


**Figure S1.** Single crystal structure of the NdFeB magnetic nanoparticle used in this study.


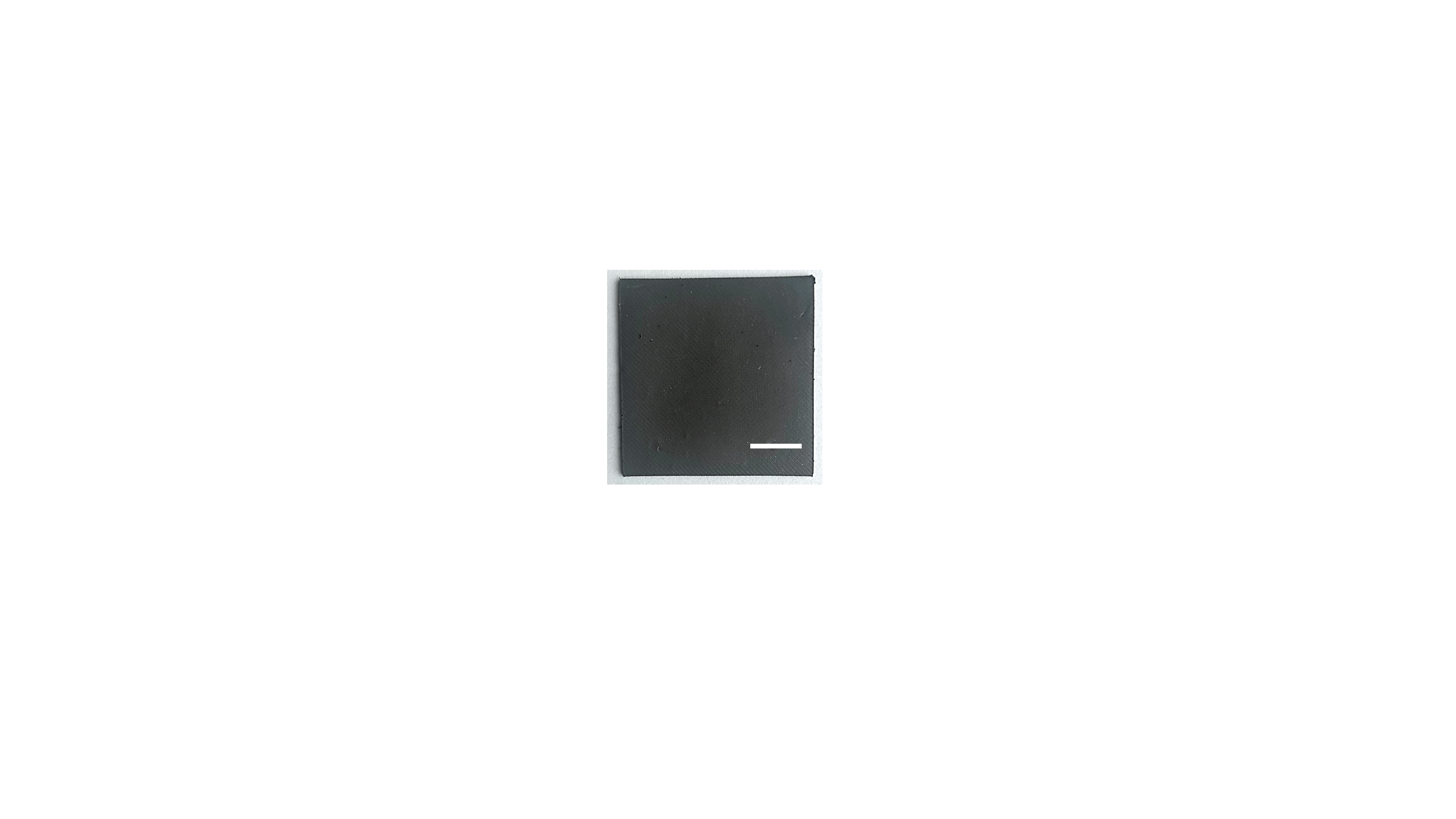


**Figure S2. Photo of the as-fabricated MC layer.** The MC layer is fabricated into a 3cm-by-3cm square-shaped membrane with different thicknesses. Scale bar,1 cm.


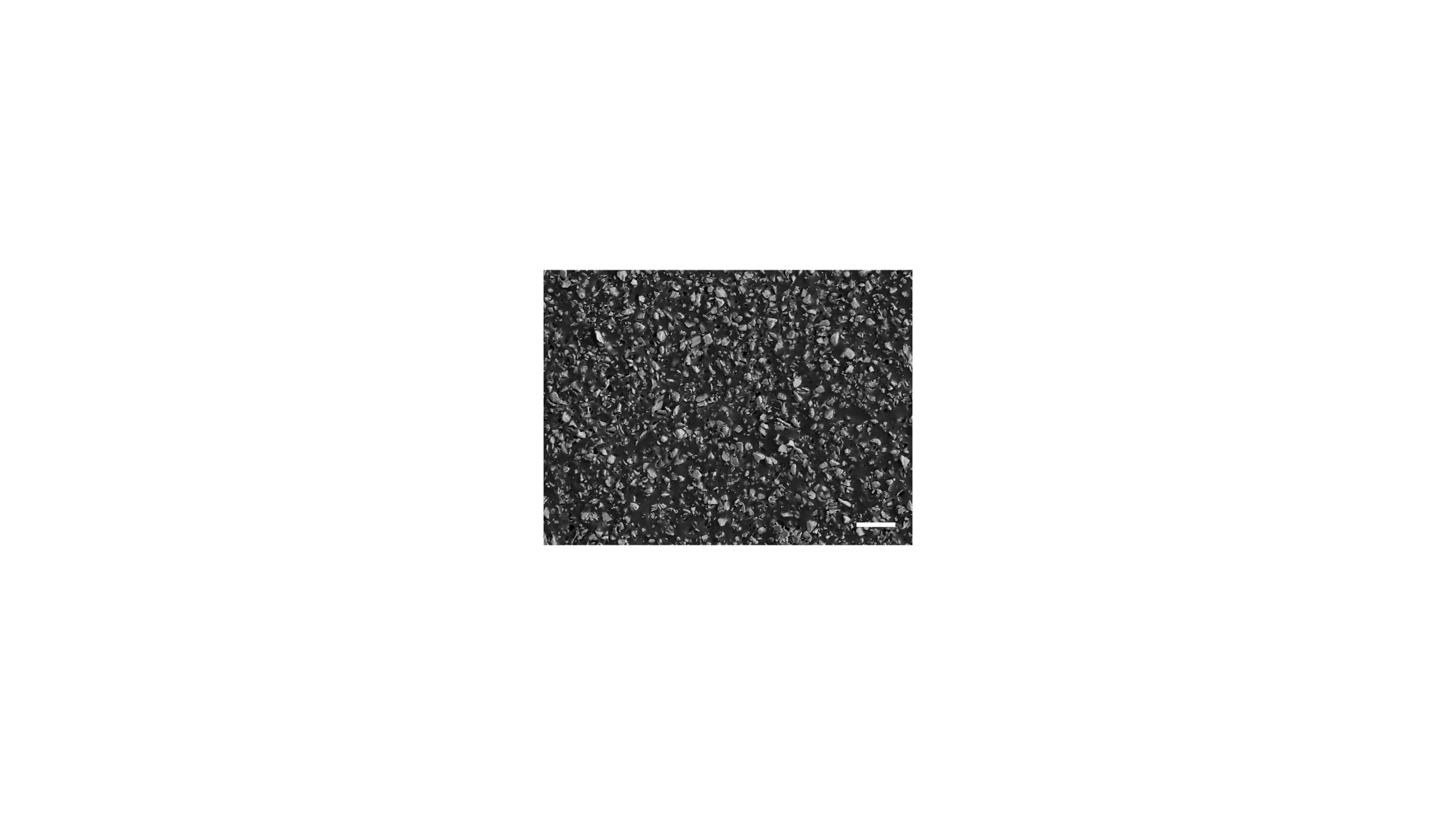


**Figure S3.** Scanning electron microscope (SEM) image of the MC layer with NdFeB magnetic nanoparticles with an average size of around 5 μm. Scale bar, 20 μm.


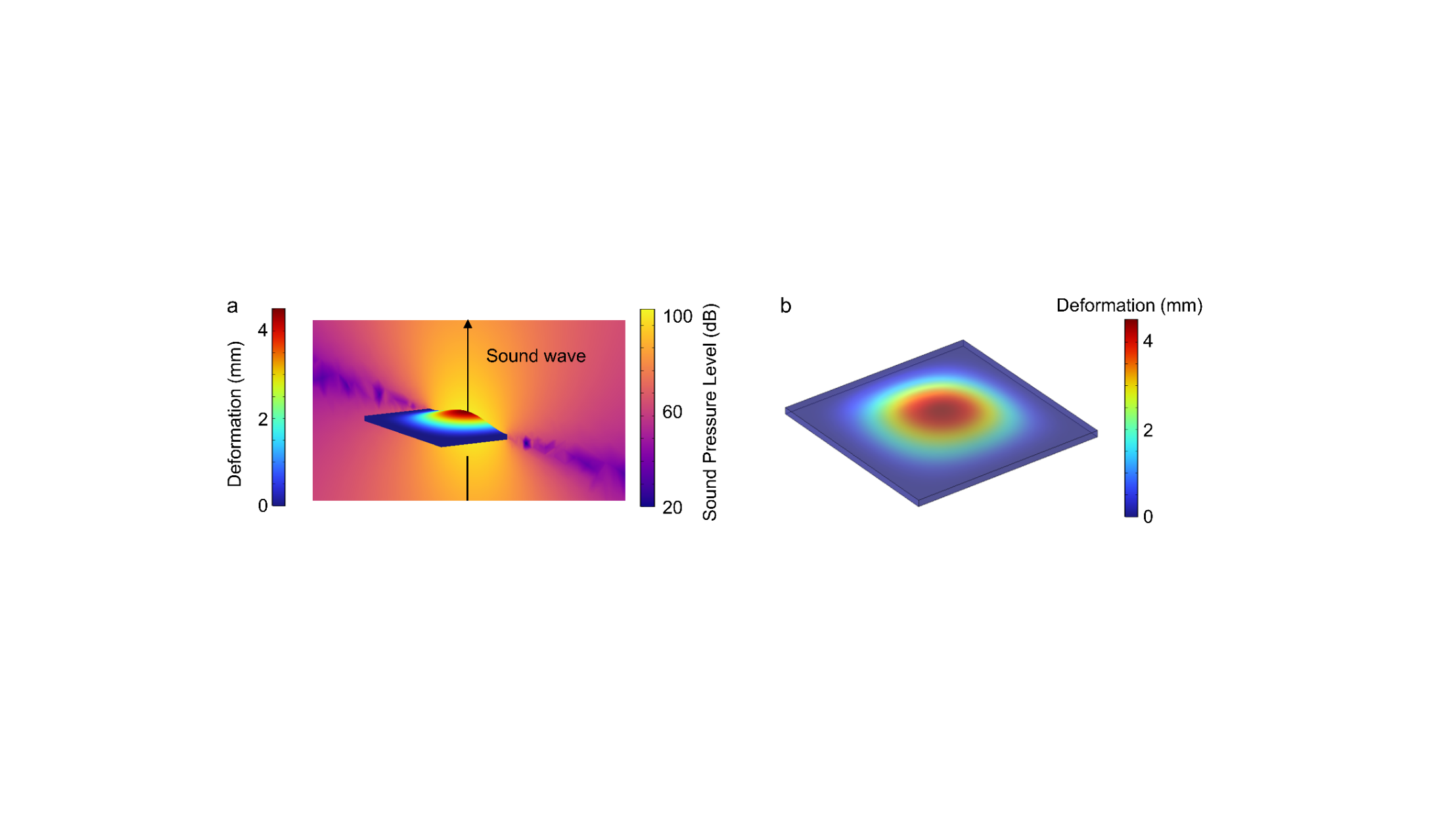


**Figure S4. COMSOL simulation of the sound wave passing through the membrane MEG.** **a**, A linear sound wave passes through the center of the membrane MEG and generates a corresponding deformation. **b**, An enlarged view of the deformation generated in the membrane of up to 4mm.





**Figure S5. Magnetic field variation of the MC layer under different stresses.** The MC layer was made with different amounts of nanomagnetic particles with a mass ratio of 80%, 75%, and 70%.


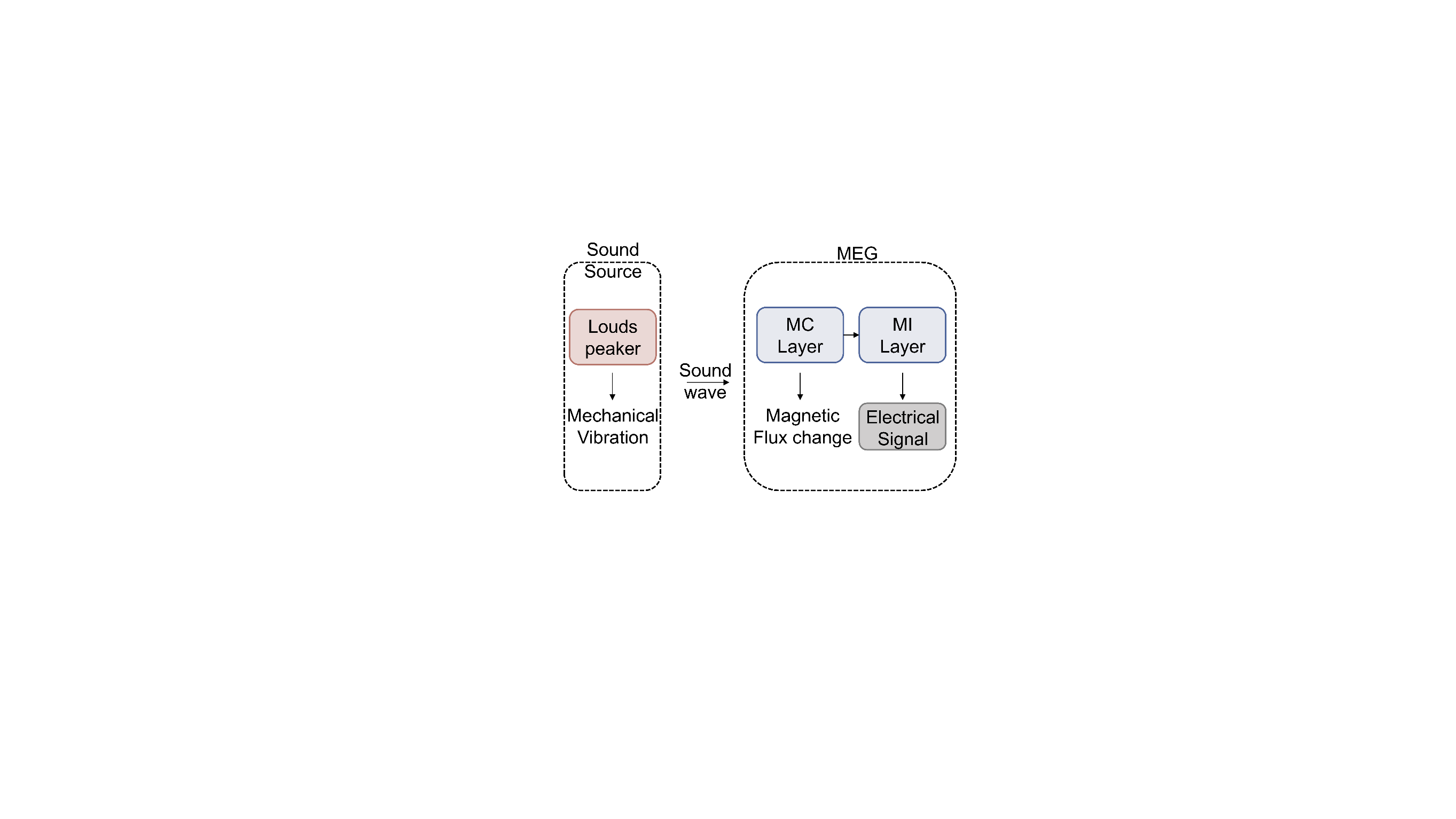


**Figure S6.** Flow chart of the testing process of the membrane MEG for acoustic energy harvesting.





**Figure S7. The current output of a membrane MEG at a thickness of 900 μm under different triggering sound frequencies**. **a**, The current output of the membrane over frequencies, the first resonance point where the current output surpasses the adjacent frequencies was indicated. b, The current outputting waveform at 60Hz. c, The current outputting waveform at 90Hz. d, The current outputting waveform at 500Hz.


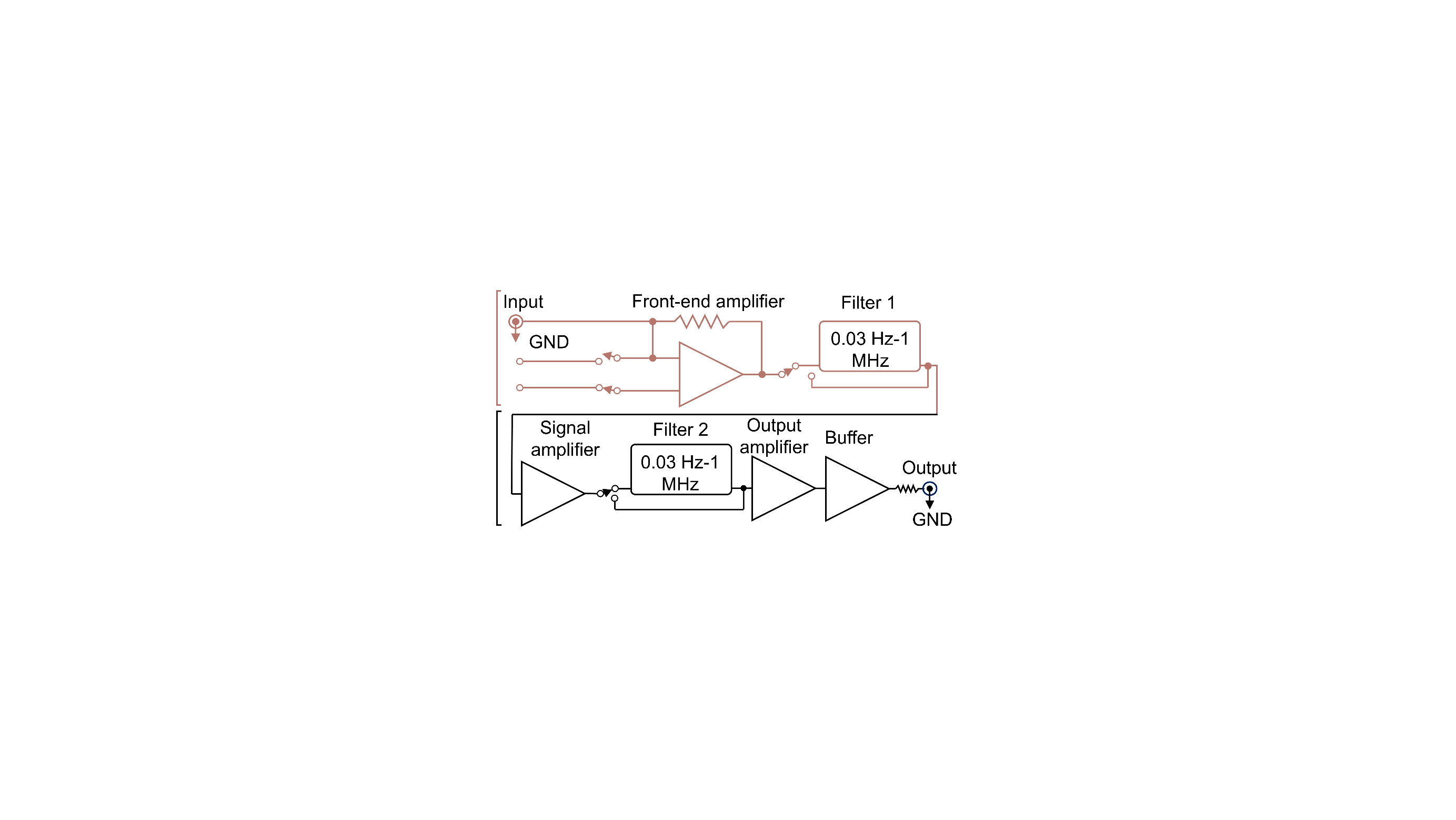


**Figure S8. Circuit design used for resonance point testing.** A band-pass filter is applied to exclude output generated at other frequencies outside the frequency the membrane MEG is being tested on.


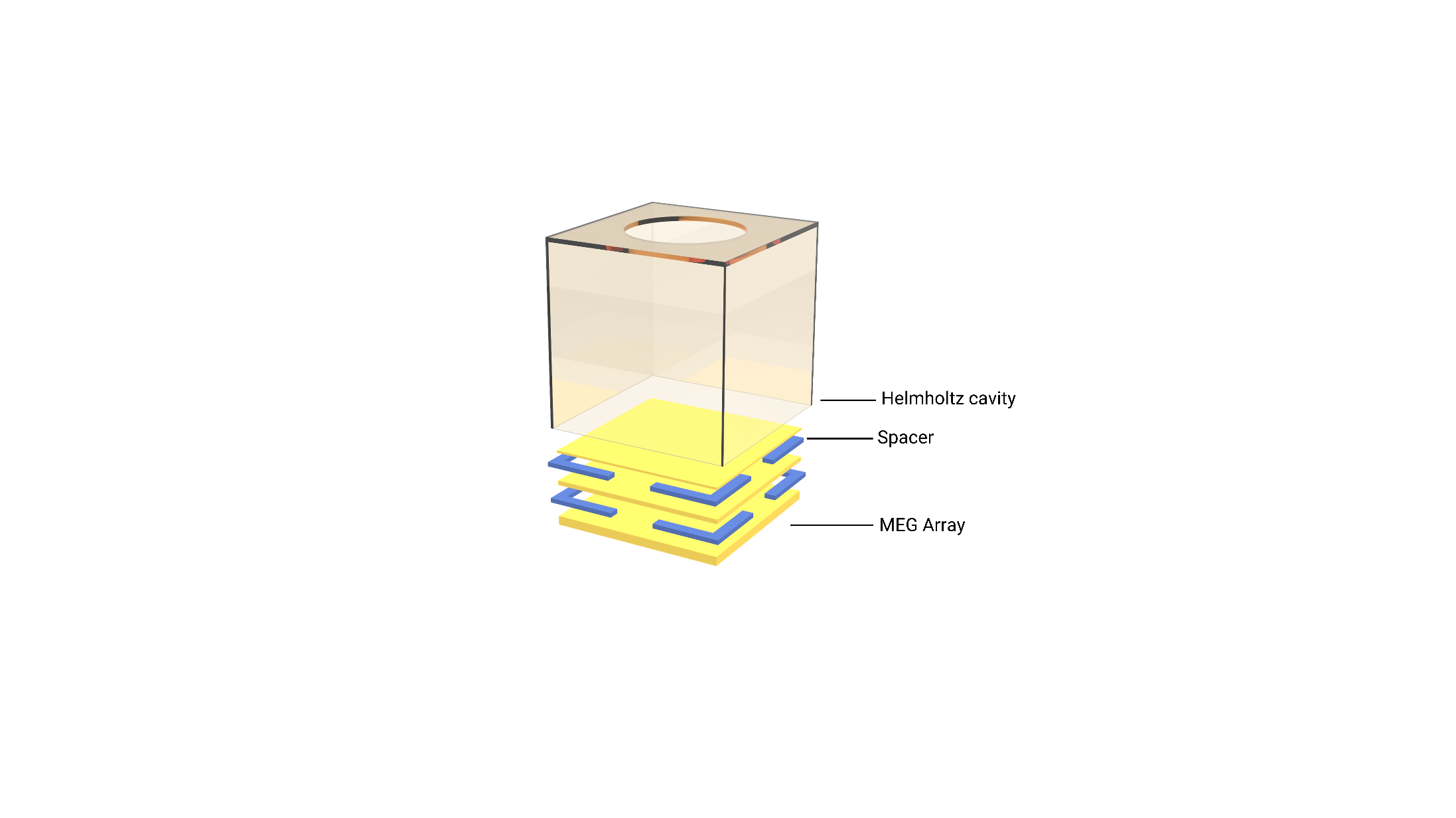


**Figure S9. The structure design of the device adopts MEG array.** Membrane MEGs with different thicknesses are stacked together with spacers to allow each membrane to vibrate freely.


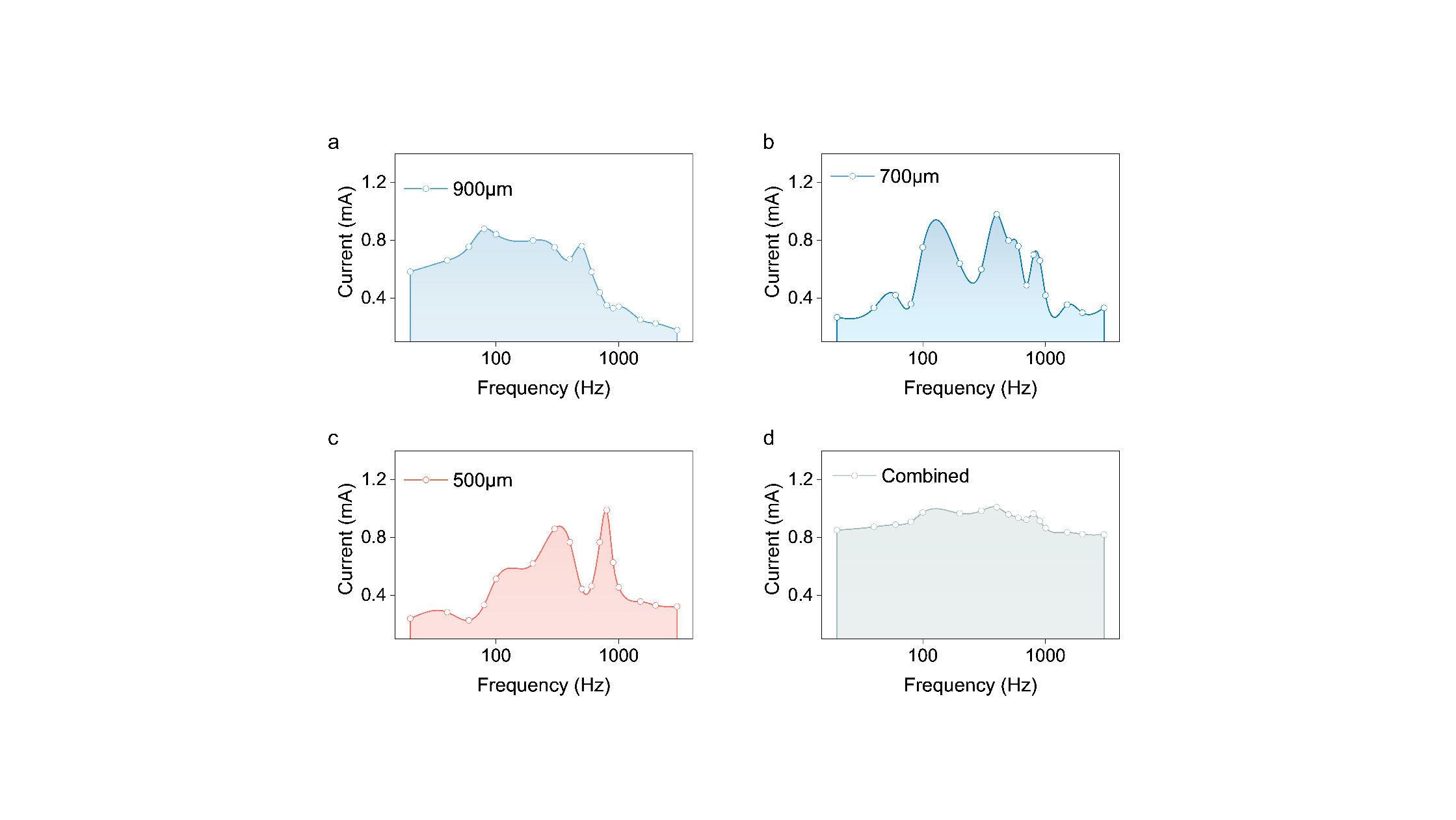


**Figure S10. Output of membrane MEGs with different thicknesses across different frequencies.** a, Outputting current of the membrane MEG with a thickness of 900 μm across frequency ranges from 20-1400 Hz. b, Outputting current of the membrane MEG with a thickness of 700 μm across frequency ranges from 20-1400 Hz. c, Outputting current of the membrane MEG with a thickness of 500 μm across frequency ranges from 20-1400 Hz. d, Outputting current of the membrane MEG array combining all three thicknesses across frequency ranges from 20-1400 Hz.


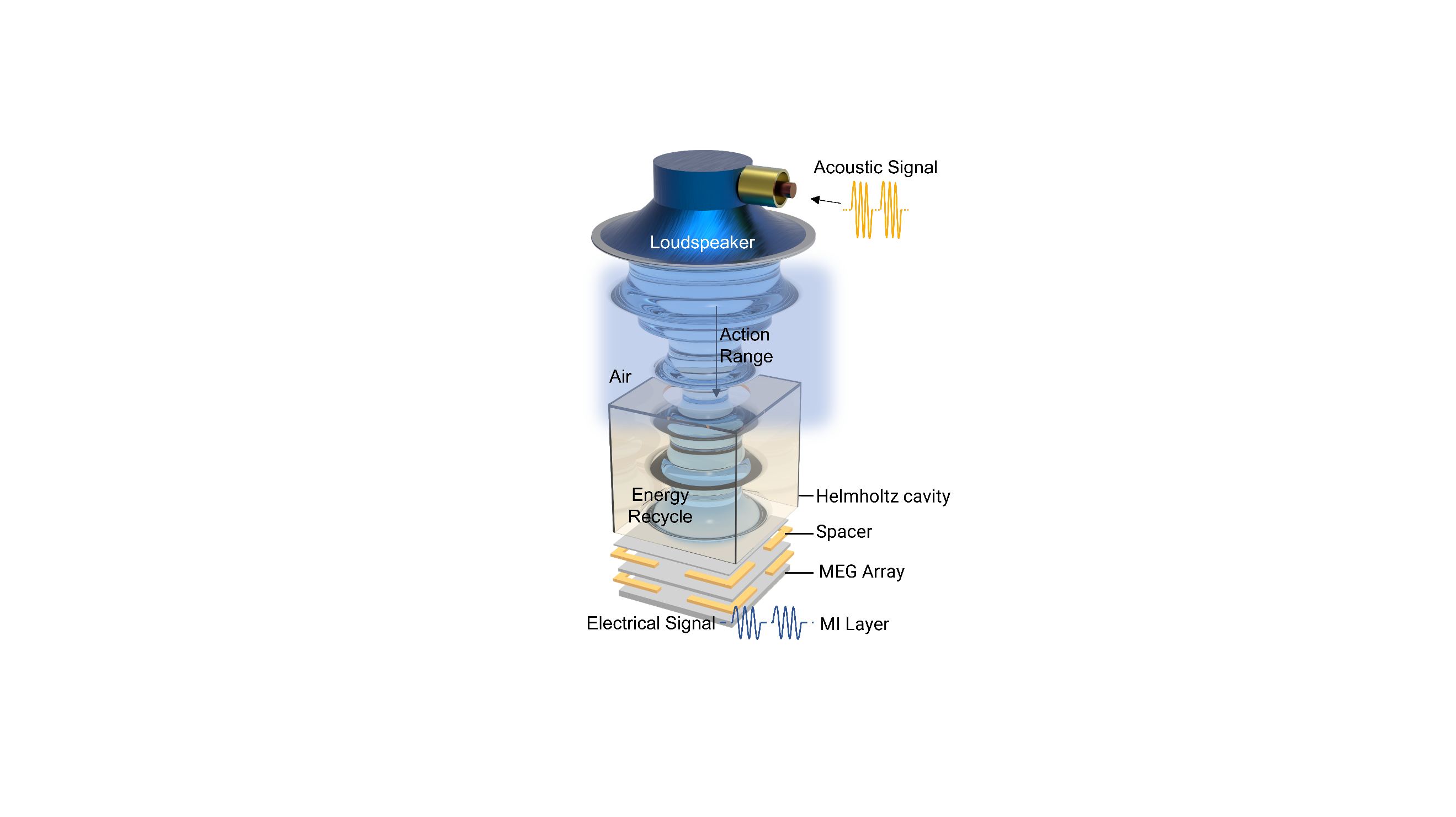


**Figure S11. Illustration of the acoustic energy harvesting performance testing setups.** The triggering sound is generated by the commercial loudspeaker travels through the air and captured by the membrane MEG, generating a corresponding current.


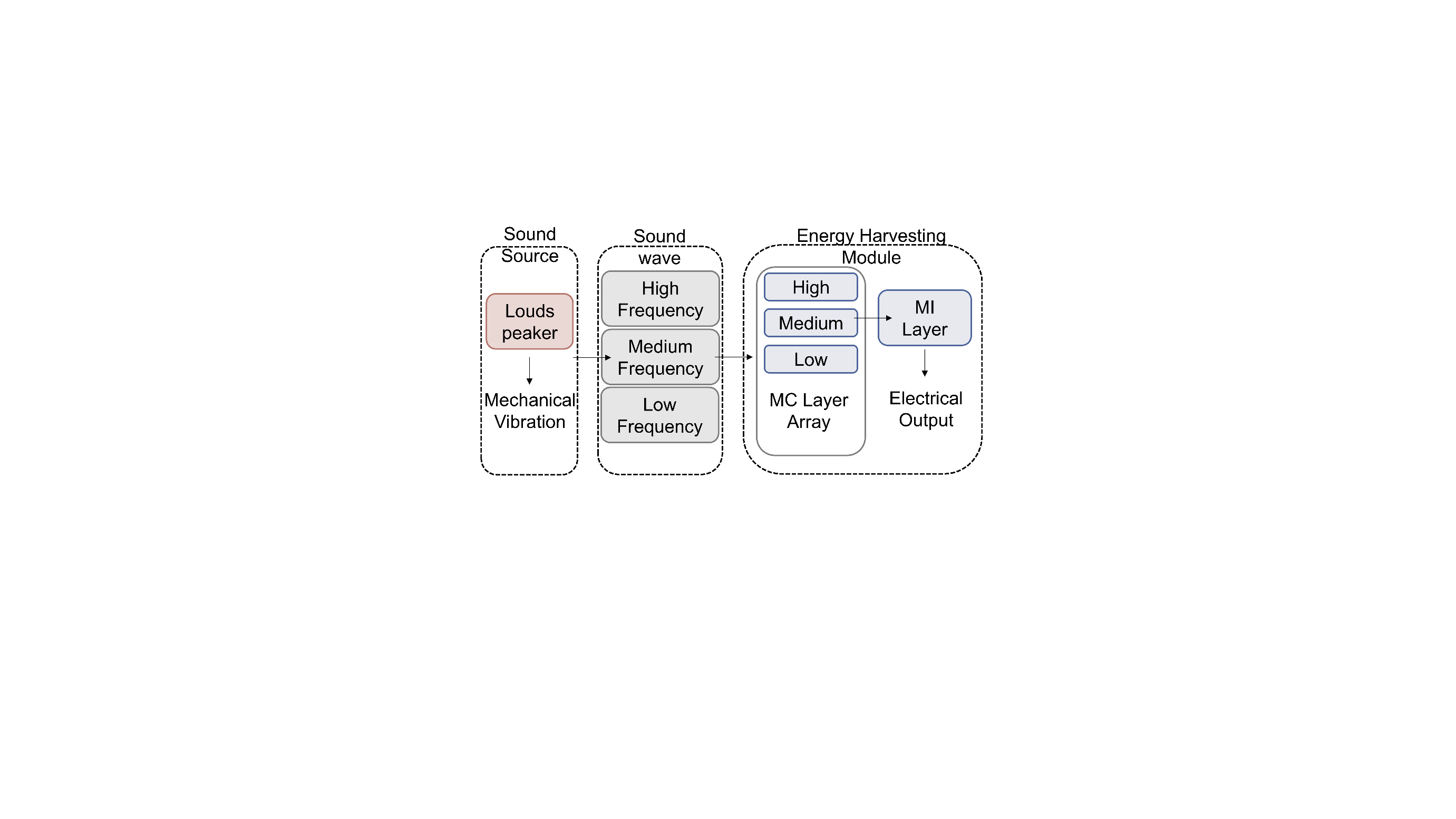


**Figure S12.** Flow chart of the testing process of the membrane MEG array.


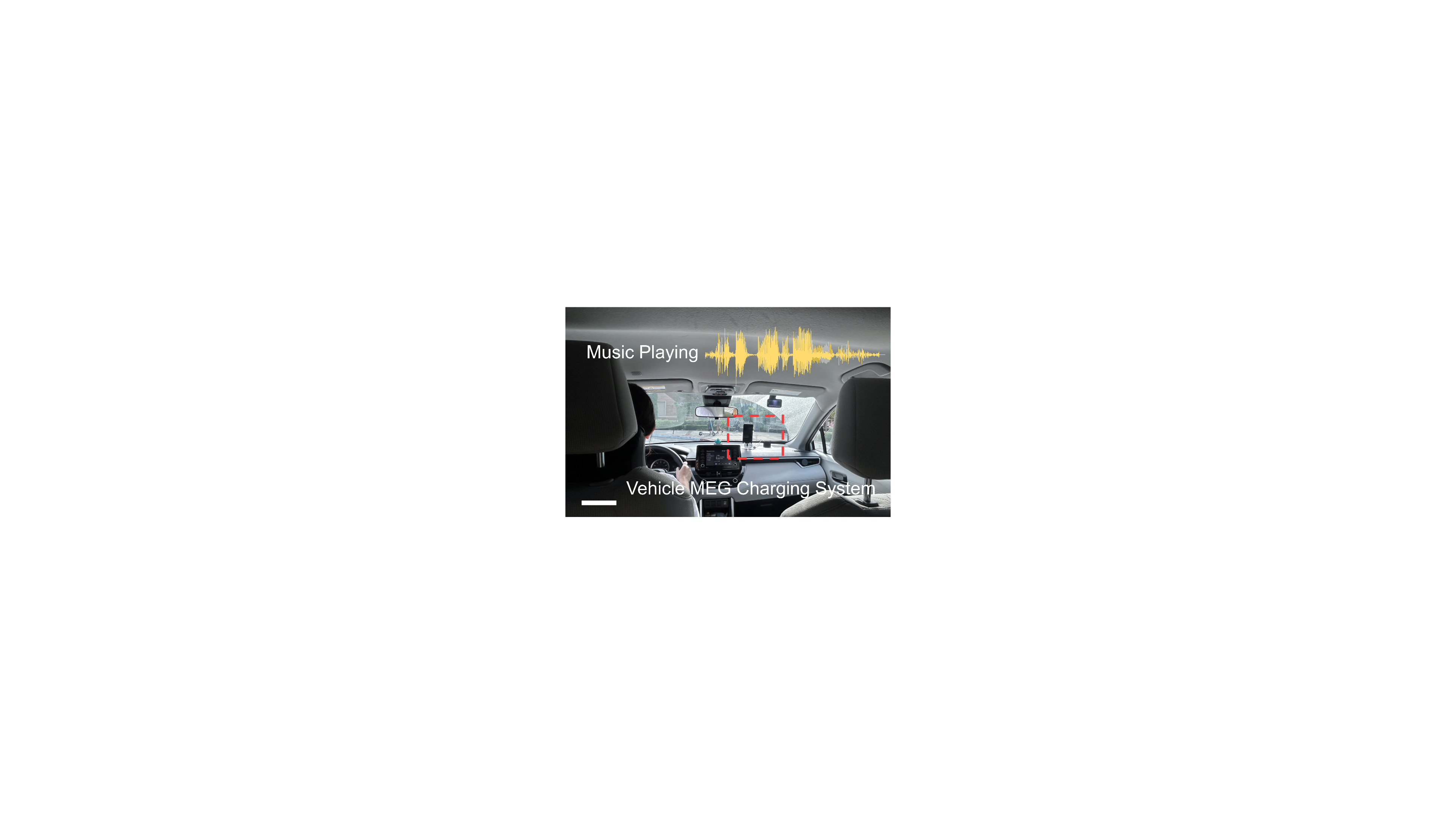


**Figure S13.** Photo of the setup of real-life scenario testing the membrane MEG in a vehicle; scale bar, 10cm.

**Table S1.** **Energy harvesting performance comparison with other literature**

| Current | Inner impedance | Stretchability | Water-proofness | Ref | Method |
| --- | --- | --- | --- | --- | --- |
| 0.97 mA | 174 Ω | Maximum 130% | Yes | This work | Magnetoelastic |
| 60 μA | 8.3 × 10^3^ Ω | - | No | 1 | Piezoelectric |
| 145 μA | 5.6 × 10^4^ Ω | - | No | 2 | Piezoelectric |
| 28.1 μA | 1.1 × 10^4^ Ω |  | No | 3 | Piezoelectric |
| 30 μA | 4 × 10^6^ Ω | - | No | 4 | Triboelectric |
| 15 μA | 600 Ω | - | No | 5 | Triboelectric |
| 15.1 μA | 1.4 × 10^6^ Ω | - | No | 6 | Triboelectric |
| 15.2 μA | 2 × 10^4^ Ω | - | No | 7 | Phononic crystal |
| 13.3 μA | 6 × 10^3^ Ω | - | No | 8 | Phononic crystal |

**Supporting references**

[1] S. N. Cha, J.-S. Seo, S. M. Kim, H. J. Kim, Y. J. Park, S.-W. Kim, J. M. Kim, *Adv. Mat.* **2010**, *22*, 4726.

[2] Y. H. Jung, S. K. Hong, H. S. Wang, J. H. Han, T. X. Pham, H. Park, J. Kim, S. Kang, C. D. Yoo, K. J. Lee, *Adv. Mat.* **2020**, *32*, 1904020.

[3] C. Lang, J. Fang, H. Shao, X. Ding, T. Lin, *Nat. Commun.* **2016**, 7, 11108.

[4] H. Zhao, X. Xiao, P. Xu, T. Zhao, L. Song, X. Pan, J. Mi, M. Xu, Z. L. Wang, *Adv. Energy Mat.* **2019**, *9*, 1902824.

[5] C. Chen, Z. Wen, J. Shi, X. Jian, P. Li, J. T. W. Yeow, X. Sun, *Nat. Commun.* **2020**, *11*, 4143.

[6] J. Yang, J. Chen, Y. Liu, W. Yang, Y. Su, Z. L. Wang, *ACS Nano* **2014**, *8*, 2649.

[7] S. Kim, J. Choi, H. M. Seung, I. Jung, K. H. Ryu, H.-C. Song, C.-Y. Kang, M. Kim, *Nano Energy* **2022**, 101, 107544.

[8] A. Allam, K. Sabra, A. Erturk, *Appl. Phys. Lett.* **2021**, 118, 103504.
